# Supplementary material for: Bioinformatics pipeline for the systematic mining genomic and proteomic variation linked to rare diseases: The example of monogenic diabetes
Source: PLoS One. 2024 Apr 18;19(4):e0300350. doi: 10.1371/journal.pone.0300350 (PMC11025945; doi:10.1371/journal.pone.0300350)
Supplement: S10 Table — (PDF) [file pone.0300350.s010.pdf]

S10 Table. Summary table of genes linked to monogenic diabetes.
In the columns of the table the information sources are listed: genes bearing variants linked to three phenotypes: “MODY”, “Monogenic diabetes” (MD), and “Neonatal diabetes” (ND) are marked in the corresponding columns. Genes with variants mentioned by Rafique et al. are marked in the corresponding column. The numbers of protein coding variants in the database of level 1 and level 2 pathogenicity confidence created in this work are given also given in the corresponding columns. The fourteen genes linked to commonly known MODY types are marked in the corresponding column with the specification of the type. The types marked with * are linked to the genes that have been reported as lacking pathogenicity and put under doubts as MODY genes by Lever et al., 2022. Finally in the last column information on the revision status by the ClinVar expert panel for the time of the manuscript submission is given.

**gene name**	**linked by ClinVar to MODY**	**linked by ClinVar to MD**	**linked by ClinVar to ND**	**found in Rafique et al.**	**included in Bonnefond et al.**	**number of variants in level 1**	**number of variants in level 2**	**common MODY type**	**status by ClinVar expert panel**
HNF1A	yes	yes	no	yes	yes	334	190	MODY3	reviewed for MODY, MD
GCK	yes	yes	yes	yes	yes	263	178	MODY2	reviewed for MODY, MD, ND
HNF1B	yes	yes	yes	yes	yes	215	161	MODY5	
ABCC8	yes	yes	yes	yes	yes	319	38	MODY12	
HNF4A	yes	yes	yes	yes	yes	81	23	MODY1	reviewed for MODY, MD, ND
INS	yes	yes	yes	yes	yes	39	22	MODY10	
KCNJ11	yes	yes	yes	yes	yes	132	19	MODY13	
INS-IGF2	yes	yes	yes	no	no	22	15		
EIF2AK3	no	yes	yes	no	yes	55	13		
PDX1	yes	yes	yes	yes	yes	32	12	MODY4	
ZFP57	no	yes	yes	no	yes	31	10		

C12orf43	yes	yes	no	yes	no	20	7		reviewed for MODY, MD
RFX6	yes	yes	yes	no	yes	25	7		
GLIS3	yes	yes	yes	no	yes	80	6		
PAX4	yes	yes	no	yes	no	23	4	MODY9*	
CEL	yes	yes	yes	no	yes	15	3	MODY8	
BLK	yes	yes	no	yes	no	12	3	MODY11*	
KLF11	yes	yes	no	yes	no	42	2	MODY7*	
NEUROD1	yes	yes	yes	yes	yes	21	2	MODY6	
FBN1	no	no	yes	no	no	3	2		
PTF1A	no	yes	yes	no	yes	22	2		
PAX6	no	no	yes	no	yes	2	2		
APPL1	yes	no	no	yes	yes	3	2	MODY14	
SLC2A2	no	yes	yes	no	yes	8	1		
LEP	no	yes	no	no	no	2	1		
SCN1A	no	no	yes	no	no	1	1		
GPR161	no	no	yes	no	no	1	1		
KCNQ1	no	no	yes	no	no	1	1		
KCNQ2	no	no	yes	no	no	1	1		
LZTR1	no	no	yes	no	no	1	1		
MAGEL2	no	no	yes	no	no	1	1		
PURA	no	no	yes	no	no	2	1		
SHANK3	no	no	yes	no	no	1	1		

FOXP3	no	yes	yes	yes	no	6	1		
TRIP11	no	no	yes	no	no	1	1		
MLKL	yes	no	no	no	no	1	1		
BSCL2	no	yes	yes	no	no	29	0		
ITGB3	no	no	yes	no	no	1	0		reviewed for ND
IGF2	yes	no	yes	no	no	0	0		
LOC102724058	no	no	yes	no	no	0	0		
LAMA2	no	no	yes	no	no	2	0		
KMT2E	no	no	yes	no	no	1	0		
TH	yes	no	yes	no	no	0	0		
INSR	yes	yes	no	yes	no	12	0		
IER3IP1	no	no	yes	no	yes	1	0		
LOC110006319	no	no	yes	no	no	0	0		
ZC2HC1B	no	no	yes	no	no	0	0		
UTRN	no	no	yes	no	no	0	0		
TRL-TAA1-1	no	no	yes	no	no	0	0		
STX11	no	no	yes	no	no	0	0		
SNORA98	no	no	yes	no	no	0	0		
SF3B5	no	no	yes	no	no	0	0		
LOC107133510	no	no	yes	no	no	0	0		
HBB	no	no	yes	no	no	1	0		
PHACTR2	no	no	yes	no	no	0	0		
LOC113939944	no	no	yes	no	no	0	0		

ASB14	yes	no	no	no	no	1	0		
CD8B	no	no	yes	no	no	0	0		
RMND5A	no	no	yes	no	no	0	0		
CD8A	no	no	yes	no	no	0	0		
RNF103-CHMP3	no	no	yes	no	no	0	0		
RET	no	no	yes	no	no	1	0		
EDEM2	yes	no	no	no	no	1	0		
PDIA6	no	no	yes	no	no	1	0		
MMP24-AS1-EDEM2	yes	no	no	no	no	0	0		
NLRP3	no	no	yes	no	no	1	0		
MT-TL1	no	no	yes	no	no	0	0		
MECP2	no	no	yes	no	no	2	0		reviewed for ND
MBL2	no	no	yes	no	no	1	0		
GATA4	yes	no	yes	no	yes	1	0		
R3HDML-AS1	yes	yes	no	no	no	0	0		
PLAGL1	no	no	yes	no	no	0	0		
LTV1	no	no	yes	no	no	0	0		
HNRNPUL2-BSCL2	no	yes	yes	no	no	0	0		
LOC123864091	no	no	yes	no	no	0	0		
LMNA	no	yes	no	yes	no	6	0		
LOC122094844	no	yes	no	no	no	0	0		

LEPR	no	yes	no	no	no	13	0		
WFS1	yes	yes	yes	yes	yes	58	0		
KCNH2	no	yes	no	no	no	1	0		
HADH	no	yes	no	yes	no	9	0		
SHLD2	no	yes	no	no	no	2	0		
GLUD1	no	yes	no	no	no	3	0		
GLIS3-AS1	no	yes	yes	no	no	0	0		
GATA6	no	yes	no	no	yes	8	0		
AGPAT2	no	yes	no	no	no	13	0		
ALMS1	no	yes	no	no	no	69	0		
CAVIN1	no	yes	no	no	no	6	0		
CAV1	no	yes	no	no	no	2	0		
GNG3	no	yes	no	no	no	0	0		
MC4R	no	yes	no	no	no	9	0		
PPARG	no	yes	no	no	no	3	0		
LOC110121471	yes	no	yes	no	no	0	0		
LOC101928371	no	no	yes	no	no	0	0		
LOC123864090	no	no	yes	no	no	0	0		
LOC113146422	no	no	yes	no	no	0	0		
HYMAI	no	no	yes	no	no	0	0		
GRIN2B	no	no	yes	no	no	1	0		
FOXP1	no	no	yes	no	no	1	0		
PARK7	yes	no	no	no	no	1	0		

EFL1	no	no	yes	no	no	2	0		
PRKAG2	yes	no	no	no	no	1	0		
CSMD1	no	no	yes	no	no	0	0		
AKT2	no	no	yes	no	no	25	0		
PAX2	yes	no	no	no	no	1	0		
PLIN1	yes	yes	no	no	no	11	0		
SLC19A2	no	yes	no	yes	yes	6	0		
SIM1	no	yes	no	no	no	7	0		
PPP1R3A	no	yes	no	no	no	24	0		
